# Sex and gender matters: A sex-specific analysis of original articles published in the Wiener klinische Wochenschrift between 2013 and 2015

**DOI:** 10.1007/s00508-017-1280-1

**Published:** 2017-10-17

**Authors:** Éva Rásky, Anja Waxenegger, Sylvia Groth, Erwin Stolz, Michel Schenouda, Andrea Berzlanovich

**Affiliations:** 1 Institute of Social Medicine and Epidemiology, Universitaetsstraße 6/I, 8010 Graz, Austria; 3 Fachbereich Forensische Gerontologie, Department für Gerichtsmedizin Wien, Medizinische Universität Wien, Sensengasse 2, 1090 Wien, Austria

**Keywords:** Research, Quality improvement, (SAGER) Guideline adherence, Research quality improvement, (SAGER) guideline adherence

## Abstract

**Background:**

The variables sex and gender are significantly related to health and disease of women and men. Aiming at quality research, biomedical publications need to account for the key variables sex and gender.

**Methods:**

All original articles published in the Wiener klinische Wochenschrift between 2013 and 2015 were extracted into a database. As a result, the 195 published articles were selected for review led by the Sex and Gender Equity in Research Guidelines (SAGER) by the European Association of Science Editors (EASE). The slightest indications of mentioning sex and/or gender were assessed by two reviewers independently from one another.

**Results:**

Of the 195 publications 4 specified sex and/or gender in the title, and 62 in the abstract. None of the authors reported whether the variables sex and/or gender may have relevance and were taken into account in the design of the study. Of the 195 publications 48 mentioned the potential implications of sex and/or gender on the study results.

**Conclusion:**

In the time span studied most of the selected articles of this journal did not account for the variables sex and/or gender systematically or adequately. For future research the existing guidelines can help authors and editors to overcome gender bias due to inadequate methods. Applying sex and gender-sensitive methods to biomedical and health research is necessary for high quality and as a precondition for results which are generalizable and applicable to both women and men.

## Introduction

In the second women’s movement in the 1970s, feminists fought for self-determined pregnancy, abortion and maternal services as a human right [1]. In the 1990s, female cardiologists published their research results on sex bias in managing coronary heart disease of women in the USA [2, 3]. As a consequence, the US National Institutes of Health (NIH) made a major commitment to research women’s health and illness in order to stop extrapolating findings from men to women. In 1994, the NIH devised sex-specific criteria to ensure equitable participation of male and female subjects in clinical studies as well as to design studies validly and meaningfully analyzing similarities and differences between the sexes [4]. In 2001, the US Institute of Medicine (IOM) released a report stating that sex must be considered in all aspects and all levels of biomedical research [5]. The demand and the endeavor to include sex and gender into medical research and health care have a history. Being aware of sex differences and devising research appropriately is thus a relatively new approach.

In recent years, significant sex differences in all fields of biomedical research were identified [6–9]. Nowadays, sex has become accepted as a fundamental determinant for the health of women and men, in addition to social determinants, such as income, occupation, education, culture, and social connectedness [10]. From this list, gender has often been omitted [11]. Gender is a non-biological determinant describing socially constructed roles, perceptions, and resources associated with being female or male in a given group or society [12]. Sex and gender interact. Their interactions are known to influence molecular and cellular processes, clinical characteristics, as well as health and disease outcomes [13]. The relevance of gender relations and sex-linked biology to a given health outcome is an empirical question; depending on the health outcome under study, both, neither, one or the other may be relevant as single, independent, or interacting determinants. Clarity of concepts, and attention to both gender relations and sex-linked biology and their interactions is critical for valid scientific research on population and individual health [14, 15].

In scientific journals gender is often mistaken for sex [16]. Robust sex, age and gender analysis of data has often been lacking in scientific publications, resulting in knowledge gaps and compromising health outcomes [17]; therefore, the design, research question, data generation, analyses and results of studies should be disaggregated by age and sex, analyzed both within sex groups as well as between sex groups, in order to understand the causal mechanisms and interactions behind health conditions and gender [18]. Randomization, blinding, and sample size calculations are recognized methods in biomedical research. Sex and gender analyses as methods influencing the quality and the generalizability of biomedical studies have not been established to the same extent [19–21]. This oversight limits the generalizability of research findings and their applicability to clinical practice, for women and for men [22].

The US NIH repeatedly addressed the issue of sex and gender inclusion across biomedical research multidimensionally, through program oversight, review and policy, as well as through collaboration with stakeholders including publishers. Several journals now require authors to specify sex and gender-related information [23, 24].

Since 2005, to improve the quality of research reporting, the International Committee of Medical Journal Editors (ICMJE) has required clinical trials to be registered as a prerequisite for publication [25]. Changing the requirements, therefore, led to a significant shift in research reporting [26]. Along this line, the Gender Policy Committee (GPC) of the European Association of Science Editors (EASE), developed a standard for sex and gender equity in scientific reporting as a basis for authors and journals to revise their policies and practices [22, 27, 28]. The Sex and Gender Equity in Research (SAGER) guidelines are a comprehensive procedure for reporting sex and gender information including the sex of research subjects, study design, data analyses, results and interpretation of the findings [22, 29]. They imply to give reasons when only women or only men were studied, to discriminate between sex and gender (mostly in human research), to analyze how sex and/or gender impact the results, and to discuss sex and gender issues when relevant [30].

## Methods

As an Austria research team we analyzed the major ranked Austrian medical journal for its sex and/or gender-specific reporting, being aware that considering these variables has not explicitly been included in its editorial policy [22]. We chose the original articles published in the Wiener klinische Wochenschrift between 2013 and 2015. This time span was selected since the international scientific discussion on sex and gender in medicine gained momentum then.

We researched and extracted all original articles published in the Wiener klinische Wochenschrift in the chosen period into a database. In a first step, we included articles denoted as reviews, consensus statements, and almanacs/overview articles. Papers focussing on children/adolescents, sex-specific diseases, such as prostate or cervical cancer, animals, and blood specimens as well as supplementary volumes were excluded at this time. The two reviewers (ÉR, AW) independently screened the selected articles for the presence of sex and/or gender applying the SAGER guidelines [22]. They encompass first, whether the authors clearly defined the variables sex and/or gender, secondly, whether the study population was specified in the title and/or in the abstract, thirdly, whether the number of women and men studied was given, fourthly, whether information on sex-disaggregated data on research outcomes was present and fifthly, whether authors debated sex/gender differences or mentioned the effects of not analyzing sex and gender. The slightest mentioning of sex/gender similarities and/or disparities was assessed by the reviewers as, “yes, the authors reflected sex/gender differentiation” (see Table 1). Finally, the two reviewers formed a consensus on discrepancies in their evaluations.

**Table 1:** Applying SAGER guidelines to publications of the Wiener klinische Wochenschrift

Sex and/or gender	Present	
	Total: 195 publications	
	Absolute numbers	Percentage
Specified in title	4	2
Specified in abstract	62	32
Data on sex of the study population	157	81
Sex-disaggregated data in results section	75	38
Sex and/or gender-specific publications cited*	75	38
Discussion section	48	24

*in addition to SAGER guidelines

## Results

A total of 255 articles were published in the journal between 2013 and 2015. From these, we analyzed 195 relevant publications altogether, 172 original articles, 11 reviews, 9 consensus reports, and 3 almanacs. Overall, very few publications clearly conceptualized sex and gender. Of the 195 publications 4 specified sex and/or gender in the title, and 62 in the abstract (see Table 1). The authors did not report in any of the publications whether sex and/or gender differences were relevant or were taken into account in the design of the study. Of the 195 publications 157 provided information on the sex of the study population. Of these articles, 62% (98) studied more males than females. In nearly 1 out of 4 of these articles, 25% (39), more than twice as many subjects were males. Disaggregated data on sex in relation to the specific research question was present in 75 of the 195 publications. Of the 195 publications 48 mentioned the potential implications of sex and/or gender on the study results. No article gave rationales for not conducting sex and gender analysis.

## Discussion

In this review we analyzed the reporting of sex and gender research results in the major Austrian medical journal over a period of 3 years. The scientific literature is replete with evidence that sex and gender have a powerful effect on the health of individual women and men and of populations at large. Nevertheless, the variables sex, and more often, gender are often overlooked in studies and in scientific reporting [31]. Women are underrepresented in clinical research, despite the US NIH guidelines [32].

Our results provide further evidence that authors and editors are still failing to follow good practice guidelines [17]. In the studied articles of the Wiener klinische Wochenschrift, sex and gender as determinants of health were mostly poorly defined. Sex-disaggregated data are provided in far less than half of the studies. This is a striking result, as it concerns issues on which evident sex differences were previously published, e. g. rheumatoid arthritis, cardiac diseases and diabetes. The generalizability and applicability of results may thus be compromised. Even when sex-specific data are provided, authors seldom discuss the possible impacts of these results. As an imperative for evidence-based research, we suggest that these relevant determinants of health should be respected [33]. We recommend the editors to use the SAGER guidelines as a lever to change practice. These first results may spur the awareness of this issue in Austria [34].

## Strengths and limitations

This is the first sex and gender-based analysis of scientific articles of an Austrian medical journal. Based on scientific research results, we assume that all investigated research questions have a sex and/or gender relevance. Nonetheless, we limited our first analysis to human research. Restriction to one journal and to the chosen time span may lead to bias.

## Conclusion

Hitherto, very few studies chose sex and gender-sensitive approaches in research reporting [35–37]. In accordance with these international results, our findings show that sex and gender analyses have yet to be applied to the publications of the major Austrian medical journal. This paper could be a baseline for future assessments of change. Applying sex and gender-sensitive methods to biomedical and health research reporting is necessary to benefit health care of women and men [38]. Authors can draw on existing tools to overcome gender bias as a result of using inadequate methods [39–41]. Editors are the key change agents in this process. They can apply this lever by adopting the SAGER guidelines. We suggest that editors adopt guidelines and prerequisites for authors, which are standard in international journals. Ultimately, such a shift in editorial policies will yield better science and thus better health outcomes by addressing sex and gender-related health inequities.

**Table 2:** Sex and Gender Equity in Research (SAGER) guidelines [22]

General principles [22]	
Authors should use the terms *sex* and *gender* carefully in order to avoid confusing both terms.	
Where the subjects of research comprise organisms capable of differentiation by sex, the research should be designed and conducted in a way that can reveal sex-related differences in the results, even if these were not initially expected.	
Where subjects can also be differentiated by gender (shaped by social and cultural circumstances), the research should be conducted similarly at this additional level of distinction.	
Recommendations per section of the article:	
Title and abstract	If only one sex is included in the study, or if the results of the study are to be applied to only one sex or gender, the title and the abstract should specify the sex of animals or any cells, tissues and other material derived from these and the sex and gender of human participants.
Introduction	Authors should report, where relevant, whether sex and/or gender differences may be expected.
Methods	Authors should report how sex and gender were taken into account in the design of the study, whether they ensured adequate representation of males and females, and justify the reasons for any exclusion of males or females.
Results	Where appropriate, data should be routinely presented disaggregated by sex and gender. Sex and gender-based analyses should be reported regardless of positive or negative outcome. In clinical trials, data on withdrawals and dropouts should also be reported disaggregated by sex.
Discussion	The potential implications of sex and gender on the study results and analyses should be discussed. If a sex and gender analysis was not conducted, the rationale should be given. Authors should further discuss the implications of the lack of such analysis on the interpretation of the results.
